# Occurrence of methicillin-resistant *Staphylococcus aureus* in farm workers and the livestock environment in Mecklenburg-Western Pomerania, Germany

**DOI:** 10.1186/s13028-014-0053-3

**Published:** 2014-08-21

**Authors:** Carmen Dahms, Nils-Olaf Hübner, Christiane Cuny, Axel Kramer

**Affiliations:** 1 University Medicine Greifswald, Institute of Hygiene and Environmental Medicine, Walther-Rathenau-Straße 49a, Greifswald, 17487, Germany; 2 Institute of Medical Diagnostic (IMD), Pappelallee 1, Greifswald, 17489, Germany; 3 Robert Koch-Institute, National Reference Center for Staphylococci and Enterococci, Burgstraße 37, Wernigerode, 38855, Germany

**Keywords:** Methicillin-resistant Staphylococcus aureus, MRSA, Animals, Humans, Livestock, Chickens, Poultry, Pig, Cattle, Zoonoses, Germany

## Abstract

**Background:**

Livestock-associated methicillin-resistant *Staphylococcus aureus* (LA-MRSA) has a wide host range and is transmissible to humans, especially to those with close contact to colonized animals. This study presents the first data on the occurrence of MRSA in farm workers and livestock farms (pig, cattle and poultry) in the federal state of Mecklenburg-Western Pomerania in northeast Germany. 78 farm workers at pig farms, cattle farms and poultry farms were tested for MRSA via pooled nasal and pharyngeal swabs. Additionally, from each of the 34 participating farms (17 pig farms, 11 cattle farms, 6 poultry farms) five dust samples were taken from the direct surroundings of the animals. Furthermore, oropharyngeal swabs were additionally taken from 10 animals per poultry farm. Isolated MRSA strains were characterized and confirmed using PCR and *spa* typing. Resistance patterns were obtained using the broth microdilution assay.

**Results:**

In total, 20 of 78 (25.6%; 95% CI:17.3-36.3) farm workers were positive for MRSA. All MRSA-positive workers were employed at pig farms. Six of 17 (35.3%; 95% CI:17.3-58.7) pooled dust samples from pig farms were also positive. Overall, six *spa* types were identified, of which t034 predominated. All strains belonged to LA-MRSA CC398 and were resistant to tetracycline. Resistance to lincosamides, macrolides, fluoroquinolones and aminoglycosides was present in some strains. Three farm workers harbored the identical *spa* type and antimicrobial resistance pattern found in the corresponding dust sample. Neither workers, dust samples from cattle and poultry farms, nor oropharyngeal poultry swabs tested positive for MRSA.

**Conclusions:**

The present study emphasizes the importance of MRSA on pig farms and pig-farm workers in the rural region of Mecklenburg-Western Pomerania, whereas LA-MRSA could not be isolated from cattle and poultry farms.

## Background

As far back as the 1960s, shortly after introduction of the β-lactam antibiotic methicillin, methicillin-resistant *Staphylococcus aureus* (MRSA) strains emerged in hospitals and care facilities. Today, MRSA is one of the most widespread nosocomial pathogens [1]. MRSA may lead to severe soft-tissue and skin infections, pneumonia and bloodstream-related infections [2]. There are three main categories of MRSA, which are discriminated by epidemiological and molecular typing: hospital-associated MRSA (HA-MRSA), community-associated MRSA (CA-MRSA) and livestock-associated MRSA (LA-MRSA). HA-MRSA has its origin in healthcare facilities and usually affects people with certain risk factors, e.g., contact with MRSA patients [3]. In contrast, CA-MRSA is found in non-hospitalized individuals without the typical risk factors. This MRSA is typically associated with clonal lineages other than HA-MRSA [3]. In general, MRSA as well as methicillin-sensitive *S. aureus* (MSSA) may harbor additional virulence genes which can complicate the progression of infection [4]. Panton-Valentine leucocidin (PVL) is one of these and may lead to necrotizing pneumonia and severe soft-tissue infection [5],[6]; it is often associated with CA-MRSA [7]. LA-MRSA is found in farm animals and people with close livestock contact. In this context, the multilocus sequence type 398 (ST398) is frequently found, especially at pig and cattle farms [8],[9]. However, it can be found in poultry, where other sequence types, e.g., ST5 and ST9, are also common [10]–[12]. Livestock are mostly asymptomatic carriers, but infections are possible [13],[14]. There is some evidence that humans originally transferred a MSSA strain to swine. This strain may have evolved into LA-MRSA ST398, i.e., by acquiring resistance to β-lactam antibiotics and tetracyclines [15].

Farm workers are at risk of becoming carriers themselves [8],[16]–[18]. Even if ST398 may have fewer virulence-associated genes than many HA-MRSA or CA-MRSA strains [19]–[21], severe infections in humans have been reported [22],[23]. Persons with close contact (especially family members) to farm workers are at higher risk of acquiring MRSA as well, but the risk is obviously lower than in humans with direct livestock contact [24]. The number of nosocomial infections caused by LA-MRSA is not yet completely known, as sequencing of the MRSA type is not routinely performed in German hospitals. About 0.8-2% of the MRSA strains isolated in hospital settings are presumed to be livestock-associated [19].

The Commission for Hospital Hygiene and Infection Prevention (KRINKO, Kommission für Krankenhaushygiene und Infektionsprävention) at the Robert Koch Institute, Berlin, Germany, recommends screening upon admission of individuals working with livestock to avoid infections caused by and transmission of MRSA [25].

The purpose of this study is to provide a first impression of the occurrence of MRSA in farm workers and the livestock environment in Mecklenburg-Western Pomerania (MP), and compare the isolates based on resistance profiles and *spa*-typing. MP, located in northeast Germany, is the most sparsely populated German state, but is home to large-scale agricultural holdings with high average numbers of cattle and pigs per farm. For example, in 2010, MP had 4,725 agricultural holdings whereas Lower Saxony had 41,735 [26]. Nevertheless, the average numbers of pigs per farm was more than four times higher (3905 vs. 886) and for cattle more than two times higher (174 vs. 75) than nationwide averages [27].

## Methods

### Design

A cross-sectional study was conducted between March and June 2012 to assess the presence of MRSA in people who are in close contact with farm animals in comparison to MRSA occurrence on barn surfaces and in farm animals. The study was based on voluntary participation; therefore, at some farms, no workers were sampled.

In total, 78 people (31 female, 47 male) with livestock contact (76 farm workers, 2 other persons with close livestock contact) from 23 farms were sampled. Of these persons, 17 were in contact with poultry, 25 with cattle and 36 with pigs.

**Figure 1:**
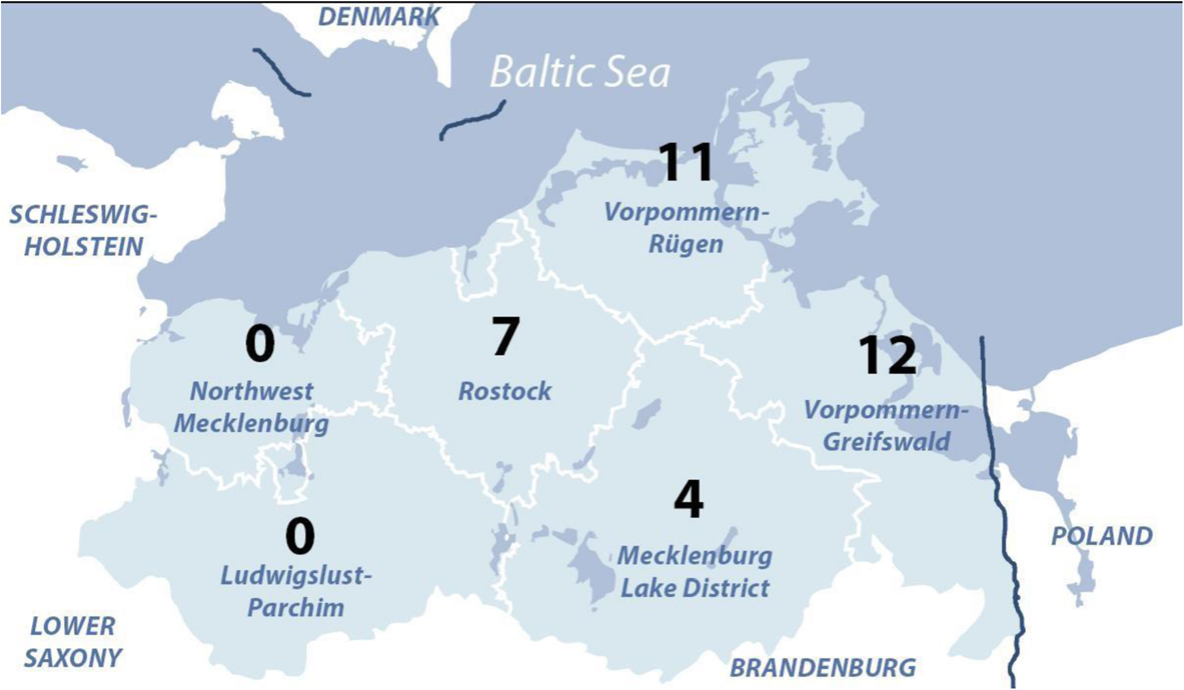
**Distribution of participating farms in Mecklenburg-Western Pomerania.** The figure shows a map of the federal state of Mecklenburg-Western Pomerania, divided into its districts Northwest-Mecklenburg, Ludwigslust-Parchim, Rostock, Vorpommern-Rügen, Mecklenburg Lake District and Vorpommern-Greifswald. The two independent cities Rostock and Schwerin are mapped, but not labelled as no farms were located in these areas. The distribution of the 34 participant farms is depicted: 11 farms were located in Vorpommern-Rügen, 12 in Vorpommern-Greifswald, 4 in Mecklenburg Lake District and 7 in the District of Rostock.

In total, dust samples were taken at 17 pig, 11 cattle, and 6 poultry farms (4 broiler farms, 2 turkey farms). The inclusion criteria were an adequate number of animals (>50 pigs or cattle; >10,000 chickens or turkeys, only for fattening) and preferentially situated in the northern part of MP (Figure 1). On poultry farms, additional oropharyngeal swabs were taken from ten randomly selected animals per farm.

The study was approved by the Ethics Committee of the University of Greifswald (No. BB07/12); Trial registration: NTR3324.

### Sampling and evaluation

All participants gave written informed consent after clarification of the purposes of the study. The participants themselves took combined nasopharyngeal samples with a dry sterile swab (Amies transport medium, Transystem®, Copan Italia *Spa*, Brescia, Italy) under supervision by the investigators. Information about age, sex, and the average daily working hours (working five days per week) of the farm workers as well as the specialization of the farm (e.g. breeding farm, dairy farm) and whether it was an organic or conventional farm were recorded. All collected data was electronically stored and evaluated using IBM SPSS Statistics 22 to compare the MRSA-positive and -negative groups for significant differences in these parameters. For calculating confidence intervals (CI) the Wilson score method was used [28].

Five dust samples per farm, each covering 500 cm^2^, were taken with dry sterile swabs (FLOQSwabs™, Copan Flock Technologies srl, Brescia Italy) based on a standard protocol [29]. The tested locations included window sills, the surface of feed fences, the surface of cattle cubicles, the surface of feed troughs and the food/water distribution system, and partition walls. A minimum of two different pens per farms were swabbed. If possible, areas with two different age groups of the animals were sampled.

On poultry farms, oropharyngeal swabs (Amies transport medium, Transystem®, Copan Italia*,* Brescia, Italy) were taken from an additional ten randomly selected animals.

All samples were stored at room temperature and were processed within 24–48 hours at a laboratory.

### MRSA isolation from humans

Swabs were streaked onto CHROMagar™ MRSA (CHROMagar, Paris, France). Additionally, the swab was shaken in Tryptic Soy Broth (Bacto™ Tryptic Soy Broth, Becton & Dickinson, Le Pont de Claix, France), incubated at 37°C for 18–24 h, and then streaked on CHROMagar™ MRSA. After 24 and 48 h, pink colonies grown on the chromogenic agar were subjected to the Slide test for *S. aureus* using Staphaurex® Plus (Remel, Dartford, United Kingdom). If positive, subcultured isolates were stored at −20°C in cryobank tubes according to the manufacturer’s instructions (Mast CRYOBANK™, Reinfeld, Germany).

### MRSA isolation of dust samples (pig, cattle or poultry) and oropharyngeal swabs

The five dust samples per farm were pooled in 100 ml Mueller-Hinton broth (Carl Roth, Karlsruhe, Germany/ heipha Dr. Müller GmbH, Eppelheim, Germany) supplemented with 6% NaCl (Carl Roth, Karlsruhe, Germany) and incubated at 37°C for 18–24 h. Each oropharyngeal swab was processed analogously. Afterwards, 1 ml was inoculated in 9 ml Tryptic Soy Broth (CASO-Bouillon, VWR International GmbH, Darmstadt, Germany and Bacto™ Tryptic Soy Broth, Becton & Dickinson, Le Pont de Claix, France) supplemented with 3.5 mg/l Cefoxitin (Sigma-Aldrich, Steinheim, Germany) and 50 mg/l Aztreonam (Sigma-Aldrich, Steinheim, Germany) and again incubated at 37°C for 18–24 h. Subsequently, 10 μl were streaked onto CHROMagar™ MRSA (CHROMagar, Paris, France) and incubated at 37°C for 24–48 h.

Putative colonies were confirmed using Staphaurex® Plus (Remel, Dartford, United Kingdom) and stored in cryobank tubes (Mast CRYOBANK™, Reinfeld, Germany).

### Antimicrobial susceptibility testing

Antimicrobial susceptibility testing was performed at the German National Reference Center for Staphylococci and Enterococci at the Robert Koch Institute (RKI), Wernigerode Branch, using the broth microdilution assay with the following substances: benzylpenicillin, oxacillin, phosphomycin, gentamicin, linezolide, erythromycin, clindamycin, tetracycline, tigecycline, vancomycin, teicoplanin, ciprofloxacin, trimethoprim/sulfamethoxazole, rifampicin, fusidic acid, mupirocin, moxifloxacin and daptomycin. For antimicrobial susceptibility testing, the recommendations of EUCAST for performance (http://www.eucast.org/antimicrobial_susceptibility_testing/) and interpretation (http://www.eucast.org/clinical_breakpoints/) were followed.

### Genotypic characterization

MRSA status was confirmed by PCR for *mec*A at the RKI [30]. *Spa* typing of the MRSA isolates was performed as described by Harmsen *et al*. [31]. All *spa* types were assigned with Ridom StaphType software version 2.2.1 (Ridom GmbH, Wurzburg, Germany). PCR for *luk*-PV was performed as described previously [32]. Multi-locus sequence typing (MLST) was performed for each representative isolate for each *spa* type according to Enright *et al*. [33]. Primers used for MLST correspond to the protocol as described previously, with the exception of the forward primer for *tpi*; we used the sequence 5-GCAT TAGCAGATTTAGGCGT-3. Assignment to sequence types was performed by means of the MLST net database (http://www.mlst.net/submissions/default.asp).

## Results

### Human samples

In total, 20 of 78 farm workers (25.6%; 95%CI: 17.3-36.3) tested positive for MRSA (Table 1). All MRSA-positive subjects worked with pigs and originated from six different farms. No differences in age and sex between MRSA-positive and negative farm workers were observed. MRSA-positive persons worked an average of 8.8 hours per day (median 8.5 hours; 95% CI: 8.3-9.2 hours) while MRSA-negative persons worked 6.9 hours per day (median 8.0 hours; 95% CI 3.2-10.6 hours). The slight differences in average daily working time were mainly caused by one MRSA-negative person working only 1.6 hours per day at the pig holding.

**Table 1:** Numbers and results of participating farm workers and farms in Mecklenburg-Western Pomerania in 2012

**Animal species**	**MRSA-positive farm workers**	**MRSA-positive farms**
Pig	20/36 (55.6% [39.6-70.5]*)	6/17 (35.3% [17.3-58.7]*)
Cattle	0/25	0/11
Poultry		
Chicken	0/8	0/4
Turkey	0/9	0/2
Total	20/78 (25.6% [17.3-36.3]*)	6/34 (17.6% [8.3-33.5]*)

*[95% CI].

### Dust samples (pig, cattle, and poultry) and oropharyngeal swabs in poultry

Six of 17 pooled dust samples (35.3%; 95% CI:17.3-58.7) from investigated pig farms were MRSA positive. MRSA-positive pig farms were found in all tested districts (Figure 1); fattening, breeding, and rearing farms were all affected. The tested pig farms included four organic farms, which tested MRSA negative. All cattle and broiler farms tested MRSA negative, as did the 60 poultry oropharyngeal swabs.

### Antimicrobial susceptibility testing

The MRSA isolates were resistant to at least two (β-lactam antibiotics, tetracycline) and a maximum of five antibiotic groups (lincosamides, macrolides, fluoroquinolones, aminoglycoside). Resistance to clindamycin (17/26) and erythromycin (16/26) was very common. To a lesser extent, resistance against ciprofloxacin (5/26), moxifloxacin (5/26) and gentamicin (3/26) was observed (Table 2). No resistance was observed against phosphomycin, linezolide, tigecyclin, vancomycin, teicoplanin, trimethoprim/sulfamethoxazole, rifampicin, fusidic acid, mupirocin or daptomycin.

**Table 2:** Typing and resistance patterns of the MRSA isolates from pig farm workers and pig farms in Mecklenburg-Western Pomerania in 2012

**Isolate**	**Clonal complex**	**MRSA** ***spa*** **type**	**Antimicrobial resistance to**						
			**OXA**	**TET**	**ERY**	**CLI**	**CIP**	**MFL**	**GEN**
Dust sample, farm 1	CC398	t1451	x	x	x	x			
Worker 4-2	CC398	t2370	x	x	x	x			
Worker 4-3	CC398	t10721	x	x					
Worker 4-4	CC398	t034	x	x	x	x			
Worker 4-5	CC398	t2370	x	x	x	x			
Worker 4-6	CC398	t2370	x	x					
Worker 4-8	CC398	t2370	x	x	x	x			
Worker 4-9	CC398	t2370	x	x	x	x			
Worker 4-10	CC398	t2370	x	x	x	x			
Dust sample farm 4	CC398	t034	x	x	x	x			
Worker 7-1	CC398	t034	x	x	x	x	x	x	
Worker 7-2	CC398	t034	x	x	x	x	x	x	
Worker 7-3	CC398	t3275	x	x	x	x	x	x	
Worker 7-5	CC398	t034	x	x	x	x	x	x	
Dust sample, farm 7	CC398	t034	x	x		x	x	x	
Worker 8-1	CC398	t034	x	x	x	x			
Worker 8-2	CC398	t011	x	x	x	x			
Dust sample, farm 8	CC398	t034	x	x	x	x			
Worker 9-1	CC398	t011	x	x	x	x			x
Worker 11-1	CC398	t011	x	x					x
Worker 11-2	CC398	t011	x	x					x
Worker 11-3	CC398	t011	x	x					
Dust sample, farm 11	CC398	t011	x	x					
Worker 12-2	CC398	t2370	x	x					
Worker 12-3	CC398	t034	x	x					
Dust sample, farm 12	CC398	t011	x	x					

All tested isolates contain the *mec*A-gene.

oxacillin (OXA), tetracycline (TET), erythromycin (ERY), clindamycin (CLI), ciprofloxacin (CIP), moxifloxacin (MFL), gentamicin (GEN).

### Typing

All isolates harbored the *mec*A gene and belonged to the clonal complex 398 (CC398), and were therefore LA-MRSA. Six different *spa* types were detected (t034, t2370, t011, t10721, t1451, t3275); t034 (9/26), t2370 (7/26) and t011 (7/26) predominated (Table 2). The other *spa* types were observed only once. None of the isolates contained PVL.

### Correlation between positive pig-farm workers and pig farms

MRSA-positive workers were found at six different farms. In five of these farms, dust samples were positive for MRSA while one MRSA-positive worker came from a farm without positive dust samples. From one farm with positive dust samples, no farmers participated. Five people working in MRSA-positive farms tested MRSA negative.

In three cases, the *spa* type and the resistance pattern found in the human isolates were identical with the ones found in the dust samples of the farms (Table 2).

## Discussion

There are already a number of reports about the colonization of farm workers with ST398 [24]. We also found a relevant proportion of MRSA-positive pig farm workers and positive dust samples in pig farms in MP, all harboring CC398. All MRSA-isolates were PVL-negative, as currently most European LA-MRSA strains are assumed to be [20]. As neither positive workers nor dust samples were found in cattle or poultry farms, at least in the region where the study was performed, the main problem seems to be in pig production.

In pig holdings, six of 17 (35.3%; 95% CI:17.3-58.7) farms showed MRSA-positive dust samples; this means that more than every third pig farm is colonized. The European Food Safety Authority (EFSA) assessed dust samples taken in European breeding pig holdings and detected a prevalence of 43.5% (95% CI:31.6-58.2) for Germany [34]. These results corroborate with our study very well. In contrast, a different study showed that 28 of 40 pig farms tested MRSA-positive in northwestern Germany [35]. The difference to our results may be due to the lack of nasal swab collection from the individual animal, but regional distinctions and the smaller sample size may also be important factors.

Detection of MRSA at three fattening farms raises the question of whether the supplier of these farms was identical, as pig trade may be a possible source for MRSA transmission [36]. However, we have no data about the origin of the fattening pigs or possible national or international suppliers (e.g. Denmark [37]). Moreover, it would be interesting to know if there were any differences in antibiotic usage between MRSA-positive and -negative farms, but these data were not accessible. Generally, restricted antibiotic use and lower animal densities may facilitate resistance prevention. There is some evidence that MRSA may be less frequent among livestock in alternative farming systems [38],[39], but studies comparing organic and conventional livestock herds are still rare. The four organic farms included in our study all tested MRSA negative. This number is too small to be representative and could be the result of pure chance, especially because some conventional farms also tested MRSA negative.

It is unclear whether colonization with LA-MRSA in humans is transient or permanent. A recent study tested the colonization rates of pig farm workers before and after a longer absence from work, and 16 of 27 of the tested farm workers remained positive after their absence [40]. Dust may also be a potential risk factor for acquiring MRSA for individuals without direct livestock contact. In a rural region in Lower Saxony, Germany, local residents who visited farms, e.g. to buy meat, had a 3.2-times higher risk (95% CI:1.4-7.4) of colonization with MRSA than did people without occupational livestock contact [41]. A correlation between exposure time and human colonization has been shown elsewhere [17], but is not indicated by the present results. However, a larger number of participants would be needed for more reliable results.

According to our results, colonization of humans with LA-MRSA seems to be common in farms harboring positive dust samples. Currently, no effective preventive measures for farm workers exist. Until very recently, the KRINKO recommended MRSA-screening of workers at pig fattening farms before hospitalization [42]. Now, this recommendation has been modified and all people with regular livestock contact are regarded as a risk population [25]. Considering our results, this is necessary since MRSA occurs in pig farms regardless of production type (breeding, rearing, fattening).

Resistance to the antibiotics tetracycline, clindamycin, and erythromycin is very common in LA-MRSA and was confirmed by the present results. Furthermore, in one pig farm, we found all MRSA isolates from the farm workers and the dust sample resistant against fluoroquinolones. Resistance to gentamicin was detected in isolates from three farm workers from two different farms (Table 2). Generally, resistance against fluoroquinolones and aminoglycosides is less common in LA-MRSA than is resistance to lincosamides and macrolides [19],[35]. Seven isolates only showed resistance against β-lactam antibiotics and tetracycline and therefore did not express multidrug resistance.

Some *spa* types and resistance patterns of the workers were identical to those found in the corresponding dust samples. This may be an indication that the farm workers acquired MRSA at work. Other MRSA isolates of workers varied in terms of *spa* types and resistance patterns, and did not match those present in the associated dust sample. It is conceivable that workers acquired the MRSA strain from animals of a previous fattening period, as other pig groups may harbor different MRSA strains. Likewise, it must be borne in mind that we analyzed pooled dust samples; nasal samples of each individual animal over a longer period of time would yield a more precise view of the existing MRSA variants at farms. Processing the dust samples separately may have facilitated the detection of different *spa* types.

The finding of MRSA-negative farm workers and dust samples at all cattle farms was surprising and could be based on different factors. MRSA is a potential colonizer of veal calves [9]; in this study, samples were not taken at exclusively veal-calf farms. However, all tested farms kept at least dairy cattle. MRSA is known as a pathogen causing mastitis in dairy cows [18],[43] and was previously found at German dairy farms [18]. Only one of 25 bulk-tank milk samples tested MRSA positive in Mecklenburg-Western Pomerania in 2009 [44]. Nevertheless, further surveillance of these farms is needed, as transmission from MRSA-positive cattle to humans may occur [9],[18],[45].

The number of sampled poultry farms was relatively small, with six farms and sixty individually tested animals. Nonetheless, the MRSA-negative outcome was not expected, as poultry often seems to be colonized. For example, Richter *et al*. found 18 of 20 tested turkey flocks to be MRSA-positive in southwestern Germany. 22 of 59 people working at these farms were MRSA-positive as well. Dust and tracheal swabs were found to be appropriate methods [46]. In contrast, Pletinckx *et al*. indicated that the combination of oropharyngeal and cloacal swabs may have been more appropriate for sampling broilers [47]. Nonetheless, the occurrence of MRSA at other poultry farms or even flocks seems probable, because MRSA has been found in poultry samples in MP in 2012 [48].

A limitation of this study includes the fact that in eleven farms (7 pig farms, 4 cattle farms), workers did not participate. Despite information on the purpose of the study and the strict data security, the farm workers or the management were unwilling to participate. In consequence, MRSA-positive workers could have been missed, particularly as in one farm with a positive dust sample, persons were not screened.

Double selective enrichment was chosen to process dust samples and poultry oropharyngeal swabs, as the assumed density of MRSA might be very small. In contrast, no selective enrichment was used for nasopharyngeal swabs from humans, as detection rates were previously shown to be at a good level [49],[50]. Nevertheless, more anatomical sample sites including, for instance, the perineum, could have led to higher detection rates [51],[52].

Due to the small sample size, the regional concentration, and voluntary participation of the farms, the study is not meant to be representative for the overall LA-MRSA prevalence in MP. Nevertheless, it reveals that LA-MRSA is an issue of interest for pig farmers and pig farms in northeast Germany. Intensive pig farming is a growing economic sector in MP and Germany is the largest pork producer in the European Union. Due to globalization, the large-scale import and export of live animals and animal products occurs daily, which emphasizes the importance of enhanced surveillance of the epidemiological situation.

## Conclusion

LA-MRSA was found in fattening, breeding, and rearing farms, with all isolates belonging to the CC398. The high colonization rate of farm workers at pig holdings in MP is worrisome and underlines the necessity of regular screening before hospitalization. Further regional and transregional surveillance of the epidemiology of MRSA in livestock and humans, preventive measures at the farm level and in the hospital sector and antibiotic stewardship are needed.

## Competing interests

The authors declare that they have no competing interests.

## Authors’ contribution

NH, CD and AK designed the study. CD coordinated and assisted with data collection and analysis and drafted the initial manuscript. NH supervised the study and the analysis of the poultry samples. CC was responsible for the antimicrobial susceptibility testing and genotypic characterization, and contributed in an advisory capacity. All authors reviewed, edited and approved the final manuscript.
